# Diagnosis of autosomal dominant polycystic kidney disease using efficient *PKD1* and *PKD2* targeted next-generation sequencing

**DOI:** 10.1002/mgg3.82

**Published:** 2014-05-23

**Authors:** Daniel Trujillano, Gemma Bullich, Stephan Ossowski, José Ballarín, Roser Torra, Xavier Estivill, Elisabet Ars

**Affiliations:** 1 Genomics and Disease Group, Bioinformatics and Genomics Programme, Centre for Genomic Regulation (CRG)Dr. Aiguader 88, 08003 Barcelona, Catalonia, Spain; 2 Universitat Pompeu Fabra (UPF)Barcelona, Catalonia, Spain; 3 Institut Hospital del Mar d'Investigacions Mèdiques (IMIM)08003 Barcelona, Catalonia, Spain; 4 CIBER in Epidemiology and Public Health (CIBERESP)Barcelona, Catalonia, Spain; 5 Molecular Biology Laboratory, Fundació Puigvert, Instituto de Investigaciones Biomédicas Sant Pau (IIB-Sant Pau), Universitat Autònoma de BarcelonaREDinREN, Instituto de Investigación Carlos III, Barcelona, Catalonia, Spain; 6 Nephrology Department, Fundació Puigvert, Instituto de Investigaciones Biomédicas Sant Pau (IIB-Sant Pau), Universitat Autònoma de BarcelonaREDinREN, Instituto de Investigación Carlos III, Barcelona, Catalonia, Spain; 7 Genomic and Epigenomic Variation in Disease Group, Centre for Genomic Regulation (CRG)Dr. Aiguader 88, 08003 Barcelona, Catalonia, Spain; 8 Dexeus Woman's Health, Hospital Universitary Quiron DexeusBarcelona, Catalonia, Spain

**Keywords:** Autosomal dominant polycystic kidney disease, genetic counseling, molecular diagnostics, targeted NGS

## Abstract

Molecular diagnostics of autosomal dominant polycystic kidney disease (ADPKD) relies on mutation screening of *PKD1* and *PKD2*, which is complicated by extensive allelic heterogeneity and the presence of six highly homologous sequences of *PKD1*. To date, specific sequencing of *PKD1* requires laborious long-range amplifications. The high cost and long turnaround time of *PKD1* and *PKD2* mutation analysis using conventional techniques limits its widespread application in clinical settings. We performed targeted next-generation sequencing (NGS) of *PKD1* and *PKD2*. Pooled barcoded DNA patient libraries were enriched by in-solution hybridization with *PKD1* and *PKD2* capture probes. Bioinformatics analysis was performed using an in-house developed pipeline. We validated the assay in a cohort of 36 patients with previously known *PKD1* and *PKD2* mutations and five control individuals. Then, we used the same assay and bioinformatics analysis in a discovery cohort of 12 uncharacterized patients. We detected 35 out of 36 known definitely, highly likely, and likely pathogenic mutations in the validation cohort, including two large deletions. In the discovery cohort, we detected 11 different pathogenic mutations in 10 out of 12 patients. This study demonstrates that laborious long-range PCRs of the repeated *PKD1* region can be avoided by in-solution enrichment of *PKD1* and *PKD2* and NGS. This strategy significantly reduces the cost and time for simultaneous *PKD1* and *PKD2* sequence analysis, facilitating routine genetic diagnostics of ADPKD.

## Introduction

Autosomal dominant polycystic kidney disease (ADPKD; OMIM IDs: 173900; 613095) is the most common inherited kidney disease, with an incidence of 1 in 400–1000 (Iglesias et al. 1983; Dalgaard and Norby 1989). ADPKD is caused by mutations in *PKD1* (16p13.3; OMIM ID: 601313) in approximately 85% of the cases (The European Polycystic Kidney Disease Consortium 1994), and in *PKD2* (4q21; OMIM ID: 173910) in the remaining 15% (Mochizuki et al. 1996). ADPKD is characterized by the development and progressive enlargement of cysts in the kidneys and other organs, eventually leading to end-stage renal disease (ESRD). The ADPKD phenotype displays a significant variability that is greatly influenced by the affected gene. Thus, *PKD1* patients have a median age at ESRD of 58 years compared to 79 years for *PKD2* mutated patients (Cornec-Le Gall et al. 2013).

Diagnosis of ADPKD is mainly performed by renal imaging such as ultrasonography, computed tomography, or magnetic nuclear resonance (Pei et al. 2009). However, molecular diagnostics is necessary in several situations: (1) when a definite diagnosis is required in young individuals, such as a potential living related donor in an affected family with equivocal imaging data; (2) in patients with a negative family history of ADPKD, because of potential phenotypic overlap with several other kidney cystic diseases; (3) in families affected by early-onset polycystic kidney disease, since in this cases hypomorphic alleles and/or oligogenic inheritance can be involved (Rossetti et al. 2009; Bergmann et al. 2011; Harris and Hopp 2013); and (4) in patients requesting genetic counseling, especially in couples wishing a preimplantation genetic diagnosis (Harris and Rossetti 2010).

Approximately 70% of the 5′ genomic region of the *PKD1* gene (exons 1–33) is duplicated six times on chromosome 16p within six pseudogenes (*PKD1P1 to PKD1P6*), which share a 97.7% sequence identity with the genuine gene (Bogdanova et al. 2001; Rossetti et al. 2012). This, together with a high GC content, the presence of many missense variants, the absence of mutation hot spots, and the high allelic heterogeneity of ADPKD, makes the molecular diagnostics of ADPKD challenging. In addition, most mutations are private variants, with a total of 1272 pathogenic *PKD1* and 202 pathogenic *PKD2* mutations reported to date (March 2014, ADPKD Database [PKDB], http://pkdb.mayo.edu). Thus, genetic diagnosis by conventional techniques of a new ADPKD family requires long-range polymerase chain reaction (LR-PCR) of the repeated region of *PKD1* followed by nested PCRs (Rossetti et al. 2002), combined with Sanger sequencing of all 46 *PKD1* and 15 *PKD2* exons. When pathogenic mutations are not identified by Sanger sequencing, multiplex ligation-dependent probe amplification (MLPA) analysis is also performed to identify potential insertions and deletions.

Therefore, there is a demand for more simple and cost-effective molecular approaches that could be used for routine diagnosis, especially now with the coming specific therapies that will require differential genetic diagnosis (Torres and Harris 2006). To address these challenges, we have developed and validated an assay that couples genome partitioning and next-generation sequencing (NGS), to comprehensively perform in one-step mutation screening in *PKD1* and *PKD2*, as an alternative to cumbersome conventional genetic testing methods.

## Material and Methods

### Subjects

High-quality genomic DNA from 53 unrelated patients was obtained from blood lymphocytes, using standard protocols. The validation cohort included 36 ADPKD patients and five control individuals that had previously undergone conventional genetic diagnosis by Sanger sequencing of all *PKD1* and *PKD2* exons and, if negative, MLPA was also applied. The discovery cohort consisted of 12 ADPKD consecutive patients received for genetic diagnosis for which no mutations were known. ADPKD diagnosis was based on standard clinical and imaging criteria. Blood samples were obtained from other family members if they were available. All samples were codified and bioinformatics mutation analysis was blindly performed. Signed informed consent was obtained for all participants. This study was approved by the institutional review board.

### Capture and multiplexed sequencing of the *PKD1* and *PKD2* genes

To carry out DNA capture, we designed a custom NimbleGen SeqCap EZ Choice Library (Roche, Inc., Madison, WI) to target the complete genomic sequence of the *PKD1* and *PKD2* genes, and 1 kb of genomic sequence flanking at the 5′ and 3′ ends of each gene, accounting for 121,322 bp. Our design also included probes to target additional genes related to other human inherited diseases, for a total of 2.1 Mb of captured DNA after removal of repetitive sequences. DNA probes were selected using the most stringent settings for probe design (uniqueness tested by Sequence Search and Alignment by Hashing Algorithm [SSAHA]) (Ning et al. 2001). However, in order to be able to generate capture probes for the duplicated *PKD1* regions, we altered the settings for probe design of this specific region to allow probes to have up to 10 close matches in the genome. No probe redundancy was allowed in the final capture design for the rest of target regions. The Browser Extensible Data file of captured regions is available on request to the authors.

Libraries were prepared with the TruSeq DNA Sample Preparation Kits (Illumina, Inc., San Diego, CA). Genomic capture from pooled libraries was carried out using NimbleGen SeqCap EZ Library (Roche, Inc.) following User's Guide v3.0 instructions, as previously described (Trujillano et al. 2013). The libraries of the patients of the validation cohort and the five controls were prepared and sequenced together with seven samples of other diseases using the same capture design and enrichment protocol in two pools of 24 samples, for a total of 48 samples multiplexed in two HiSeq 2000 (Illumina, Inc.) lanes to generate 2 × 100 bp paired-end reads. The 12 patients of the discovery cohort were enriched in a single capture reaction and were sequenced in a Miseq (Illumina, Inc.) run to generate 2 × 250 bp paired-end reads.

### Bioinformatics analysis and mutation identification and classification

The resulting fastq files were analyzed with an in-house developed pipeline previously described (Trujillano et al. 2013). All the bioinformatics tools used in this study were run using default settings unless stated otherwise. For the patients included in this study, only the sequencing data produced for *PKD1* and *PKD2* were analyzed, as stated in the signed informed consent. The reference sequences used were NM_001009944.2 for *PKD1* and NM_000297.2 for *PKD2*. In order to identify pathogenic mutations that could cause ADPKD, we applied the following cascade of filtering steps (Walsh et al. 2010):

1. We required all candidate variants on both sequenced DNA strands and to account for ≥20% of total reads at that site in order to filter out spurious variant calls caused by misaligned reads in the duplicated region of *PKD1*.
2. Common polymorphisms (≥5% in the general population) were discarded by comparison with dbSNP 137, the 1000G, the Exome Variant Server (http://evs.gs.washington.edu), and an in-house exome variant database to filter out both common benign variants and recurrent artifact variant calls, especially in the duplicated *PKD1* regions. However, since these databases also contain known disease-associated mutations, all detected variants were compared to gene mutation databases (The Human Gene Mutation Database [HGMD], http://www.hgmd.cf.ac.uk and ADPKD Database [PKDB], http://pkdb.mayo.edu).
3. Mutations that could give rise to premature truncated proteins, that is, stop mutations, exonic deletions/insertions, and large genomic rearrangements were classified as definitely pathogenic.
4. Missense and noncanonical splicing variants were considered a priori Unclassified Sequence Variants (UCV) and their potential pathogenicity was evaluated using an in silico scoring system developed for *PKD1* and *PKD2* genes as previously described (Rossetti et al. 2007). This scoring system takes into consideration a number of in silico predictors (Grantham 1974; Tavtigian et al. 2006; Rossetti et al. 2007) and population data. We scored each of these factors, the sum of which resulted in an overall Variant Score (VS). The UCV were classified into four groups: highly likely pathogenic (VS ≥ 11); likely pathogenic (5 ≤ VS ≤ 10), indeterminate (0 ≤ VS ≤ 4), and highly likely neutral (VS ≤ −1) (Rossetti et al. 2007).

We considered to be pathogenic mutations those sequence variants predicted to result in a truncated protein (classified as definitely pathogenic) and those not found in healthy controls, that segregated with the disease in families and expected to severely alter the protein sequence using in silico predictors (classified as highly likely pathogenic and likely pathogenic variants).

If no pathogenic mutations were identified, the bioinformatics pipeline automatically reported the target sequences that presented low or inexistent sequence coverage. These regions were screened by Sanger sequencing since they were more likely to contain the pathogenic variants missed by our NGS approach. Validation of newly identified single-nucleotide variants (SNVs) was performed by Sanger sequencing.

## Results

### *PKD1* and *PKD2* enrichment

Eighty-one percent of the *PKD1*- and *PKD2*-targeted bases could be covered with capture baits for a final targeted region of 98,524 bp divided into 99 individual regions, with lengths ranging from 65 to 6,493 bp (average of 995 bp) (Table S1). Noteworthy, 100% of all coding sequences, that is, the complete 46 and 15 exons of *PKD1* and *PKD2*, respectively, were covered by capture baits. The target regions that precluded bait tilling correspond only to intronic and intergenic sequences.

### Sequencing statistics

**Figure 1:**
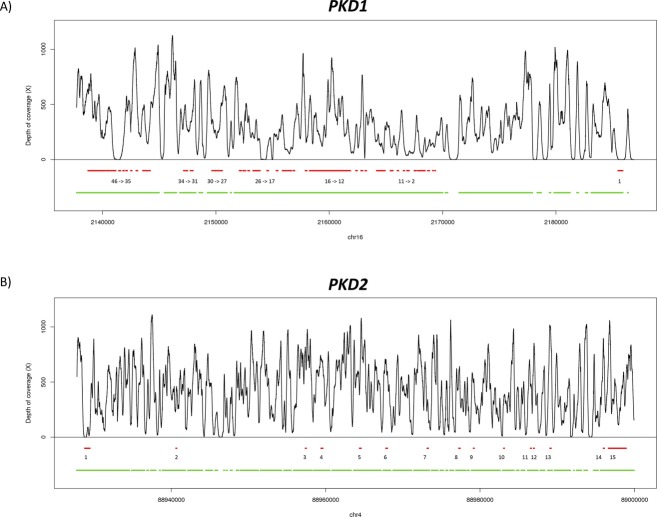
Representation of the average depth of coverage of *PKD1* (A) and *PKD2* (B) in the validation cohort. Red lines and the numbers underneath represent the exons of the genes. Green lines represent the regions tilled by capture baits.

In the validation cohort, an evenly distributed mean depth of coverage of 331X and 481X for *PKD1* and *PKD2* was achieved, respectively, on average across samples (Table 1). We achieved a sequencing depth of 289X for the 46 exons of *PKD1* and 453X for the 15 exons of *PKD2*, on average across samples (Table S2). Ninety-five percent of the coding base pairs of *PKD1* and 94% of *PKD2* were covered by more than 20 reads, which is enough for an accurate detection of known and novel mutations. Only exons 1 and 42 of *PKD1* and exon 1 of *PKD2* were not captured and sequenced at an adequate read depth (Fig. 1).

**Table 1:** Average sequencing quality control and coverage statistics of *PKD1* and *PKD2* in the validation and discovery cohorts

	Validation		Discovery	
		
Cohort	Average	SD	Average	SD
QC-passed reads	14452006.67	2252761.13	1303016.25	293339.48
Mapped	14328976.12	2236282.41	1002567.63	269009.02
Properly paired	14140971.70	2203337.48	780154.25	265250.15
*PKD1* mean coverage (X)	331.14	89.20	80.60	13.60
% *PKD1* target bases covered = 0X	1.98	0.35	3.70	0.39
% *PKD1* target bases covered ≥ 1X	98.02	0.35	98.15	0.20
% *PKD1* target bases covered ≥ 20X	95.54	1.98	86.69	2.59
% *PKD1* target bases covered ≥ 50X	92.40	4.13	65.03	4.76
% *PKD1* target bases covered ≥ 100X	84.78	6.84	52.01	1.65
*PKD2* mean coverage (X)	480.73	87.98	174.22	28.72
% *PKD2* target bases covered = 0X	0.36	0.11	0.90	0.20
% *PKD2* target bases covered ≥ 1X	99.64	0.11	99.55	0.10
% *PKD2* target bases covered ≥ 20X	99.19	0.20	98.75	0.31
% *PKD2* target bases covered ≥ 50X	98.68	0.37	92.74	3.46
% *PKD2* target bases covered ≥ 100X	97.10	2.22	67.51	8.70

Due to the lower throughput of the MiSeq sequencer, the average coverage achieved in the discovery cohort was of 81X and 174X for *PKD1* and *PKD2*, respectively, across the 12 samples (Table 1). For a comprehensive summary of the obtained sequencing results, see also Tables S3, S4.

### Detection of *PKD1* and *PKD2* mutations in the validation cohort

**Figure 2:**
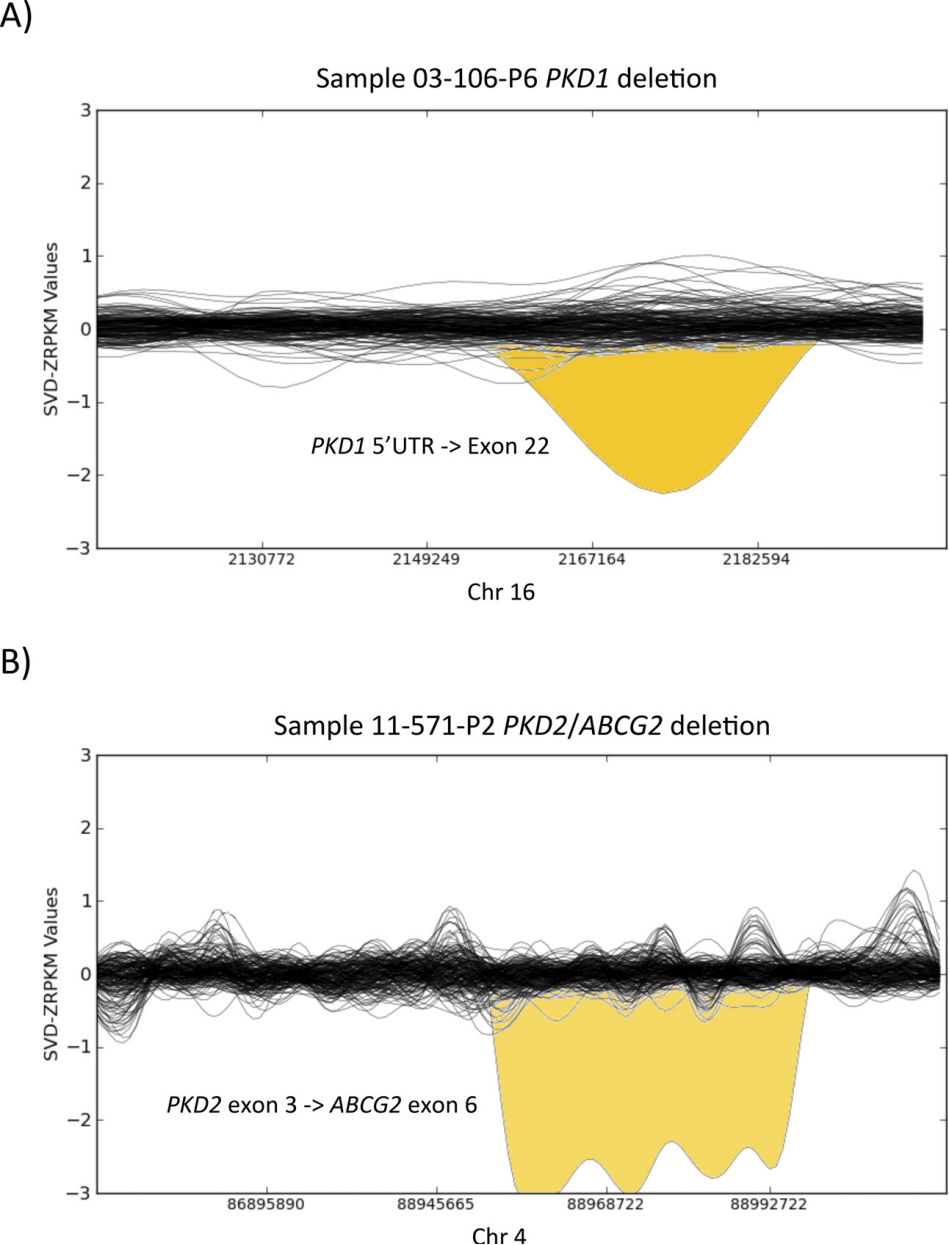
Detection of large deletions in the *PKD1* and *PKD2* genes by normalized depth of coverage analysis. Representation of the SVD-ZRPKM values calculated by Conifer for the 36 samples and 5 controls of the validation cohort. Yellow peaks indicate the two large deletions identified in this study. (A) Sample 03-106-P6 *PKD1*′s deletion (g.2154344-2186386del). (B) Sample 11-571-P2 *PKD2/ABCG2*′s deletion (g.88952828-89050618del).

For the validation cohort we selected samples with as many different types of *PKD1* and *PKD2* mutations as possible, including SNVs, short insertions and deletions (InDels), and large structural variants (SVs). We identified 35 out of 36 previously known different pathogenic mutations (30 in *PKD1* and five in *PKD2*) in their correct heterozygous state (Table 2). These results would have led to a diagnostic rate of 97.2%. Noteworthy, 25 (70%) of these mutations were spread along different exons within the segmentally duplicated regions of the *PKD1* gene, highlighting the robustness of our approach even for genes with highly homologous pseudogenes. In addition, two previously known large deletions were correctly detected. Concretely, patient 03-106-P6 presented *PKD1* g.2154344-2186386del (Fig. 2A), and patient 11-571-P2 presented *PKD2* g.88952828-89050618del (Fig. 2B). For the unique patient with a previously known mutation not identified by our NGS assay, 03-393-P3, manual inspection of the sequence alignment files revealed that the missing p.(Met1fs) was localized in a region of exon 1 of *PKD1* for which no NGS reads were available due to problems with the genomic capture.

**Table 2:** ADPKD mutations in *PKD1* and *PKD2* identified in the 36 samples of the validation cohort

Sample	Gene	Duplicated region	cDNA change	Protein change	PKDB	# Patients	Classification	Ref counts	Variants counts
03-106-P6	*PKD1*	Yes	c.1-?_8161+?del	p.(Met1fs)	Absent	0	Definitely pathogenic	–	–
12-331-P1	*PKD1*	Yes	c.566C>G	p.(Ser189*)	Present	1	Definitely pathogenic	188	62
12-382-P1	*PKD1*	Yes	c.736_737del	p.(Ser246fs)	Absent	0	Definitely pathogenic	19	14
04-016-P6	*PKD1*	Yes	c.1831C>T	p.(Arg611Trp)	Present	1	Likely pathogenic	25	13
12-235-P1	*PKD1*	Yes	c.2329C>T	p.(Gln777*)	Absent	0	Definitely pathogenic	59	37
12-366-P1	*PKD1*	Yes	c.2478delC	p.(Ile827 fs)	Absent	0	Definitely pathogenic	114	83
12-010-P1	*PKD1*	Yes	c.4888C>T	p.(Gln1630*)	Present	1	Definitely pathogenic	136	114
02-010-P6	*PKD1*	Yes	c.6583_6589del7	p.(Cys2195fs)	Present	1	Definitely pathogenic	74	79
10-326-P3	*PKD1*	Yes	c.6778_6780delATT	p.(Ile2260del)	Present	1	Highly likely Pathogenic	190	148
11-220-P2	*PKD1*	Yes	c.6221delA	p.(Asn2074fs)	Absent	0	Definitely Pathogenic	123	113
11-247-P7	*PKD1*	Yes	c.6384C>A	p.(Asn2128Lys)	Absent	0	Highly likely pathogenic	181	131
12-161-P1	*PKD1*	Yes	c.6586C>T	p.(Gln2196*)	Present	1	Definitely pathogenic	86	50
11-517-P1	*PKD1*	Yes	c.6736C>T	p.(Gln2246*)	Present	1	Definitely pathogenic	185	124
11-525-P2	*PKD1*	Yes	c.6827T>C	p.(Leu2276Pro)	Absent	0	Highly likely pathogenic	280	245
10-388-P3	*PKD1*	Yes	c.8161+1G>C	p.(?)	Absent	0	Definitely pathogenic	21	25
11-468-P1	*PKD1*	Yes	c.8251C>T	p.(Gln2751*)	Absent	0	Definitely Pathogenic	92	92
12-363-P1	*PKD1*	Yes	c.8285delT	p.(Ile2762fs)	Absent	0	Definitely Pathogenic	120	89
10-463-P3	*PKD1*	Yes	c.8311G>A	p.(Glu2771Lys)	Present	18	Highly likely pathogenic	58	63
11-457-P2	*PKD1*	Yes	c.8858A>G	p.(Asn2953Ser)	Absent	0	Highly likely pathogenic	236	191
11-287-P2	*PKD1*	Yes	c.9240_9241delAT	p.(Ala3082fs)	Present	3	Definitely pathogenic	164	80
10-193-P3	*PKD1*	Yes	c.9412G>A	p.(Val3138Met)	Present	2	Likely Pathogenic	208	188
11-595-P2	*PKD1*	Yes	c.9455_9456insC	p.(Arg3152fs)	Absent	0	Definitely pathogenic	283	184
07-172-P5	*PKD1*	Yes	c.9889G>A	p.(Val3297Met)	Absent	0	Likely pathogenic	130	114
09-403-P3	*PKD1*	Yes	c.10170+25_+45del19	p.(Gln3390fs)	Present	2	Highly likely pathogenic	96	26
10-182-P3	*PKD1*	–	c.11017-10C>A	p.(Arg3672fs)	Present	7	Highly likely pathogenic	109	83
12-144-P1	*PKD1*	–	c.10847C>A	p.(Ser3616*)	Absent	0	Definitely Pathogenic	224	177
10-353-P3	*PKD1*	–	c.11359_11360del	p.(Pro3788fs)	Absent	0	Definitely pathogenic	292	203
11-256-P2	*PKD1*	–	c.11471G>T	p.(Gly3824Val)	Absent	0	Likely pathogenic	85	63
11-168-P8	*PKD1*	–	c.12004-2A>G	p.(?)	Absent	0	Definitely pathogenic	143	113
09-446-P3	*PKD1*	–	c.12031C>T	p.(Gln4011*)	Present	4	Definitely pathogenic	106	85
11-133-P8	*PKD2*	–	c.224delC	p.(Pro75fs)	Absent	0	Definitely pathogenic	41	25
11-008-P3	*PKD2*	–	c.637C>T	p.(Arg213*)	Absent	0	Definitely pathogenic	198	172
11-170-P2	*PKD2*	–	c.965G>A	p.(Arg322Gln)	Present	4	Highly likely pathogenic	396	330
12-149-P1	*PKD2*	–	c.2050_2053del4	p.(Tyr684fs)	Present	1	Definitely pathogenic	212	181
11-571-P2	*PKD2*	–	c.709-?_2907+?del	p.(Leu237_Val968del)	Absent	0	Definitely pathogenic	–	–
09-393-P3	–	–	–	–	–		–	–	–

#Patients in previous studies, NM_001009944.2 for *PKD1* and NM_000297.2 for *PKD2*

We included in this study five control individuals without personal or family history of ADPKD to determine the clinical specificity of our assay. These controls had been previously genotyped with a HumanOmni 2.5-8 BeadChip (Illumina, Inc.) and were also used to determine the analytic sensitivity of our assay to detect heterozygous and homozygous SNVs. Genotype data were available for a total of 80 and 269 sites within the targeted regions of *PKD1* and *PKD2*, respectively. Sensitivity was of 100% both for *PKD1* (20/20) and *PKD2* (103/103). Analytic specificity was 100% both for *PKD1* (60/60) and *PKD2* (166/166) (Table S5). Of note, no spurious pathogenic calls were detected in either the control individuals or the validation cohort.

### Identification of *PKD1* and *PKD2* mutations in the discovery cohort

We detected pathogenic mutations in 10 out of 12 patients carrying a total of 11 different pathogenic mutations (10 in *PKD1* and one in *PKD2*), which lead to a diagnostic rate of 83.3%. All variants were confirmed by Sanger sequencing (Table 3). Interestingly, one patient (12–444) harbored one definitively pathogenic mutation in *PKD2* and one highly likely pathogenic mutation in *PKD1*, presenting a more severe phenotype compared to the rest of the family. For the two samples in which no pathogenic variants were identified with our NGS assay, the bioinformatics pipeline proposed a list of candidate regions with low sequence coverage that were screened by Sanger sequencing and the two causal mutations were identified. Then, by manually inspecting the alignment files of the NGS reads, we realized that we had lost p.(Val2768Met) in patient 13-102 and p.(Arg4021fs) in patient 07-335 because their locations were in poorly covered areas of *PKD1* and the variant calling and filtering algorithms had discarded them as potential false positives calls. Noteworthy, no spurious pathogenic calls were reported in any of the samples of the discovery cohort.

**Table 3:** ADPKD mutations in *PKD1* and *PKD2* identified in the 12 samples of the discovery cohort

Sample	Gene	Duplicated region	cDNA change	Protein change	PKDB	# Patients	Classification	Ref counts	Variants counts
06-056	*PKD1*	Yes	c.348_352delTTTAA	p.(Asn116fs)	Present	1	Definitely pathogenic	28	20
06-122	*PKD1*	Yes	c.7204C>T	p.(Arg2402*)	Present	2	Definitely pathogenic	24	12
07-032	*PKD1*	Yes	c.8421_8422insC	p.(Ile2808fs)	Absent	0	Definitely pathogenic	22	18
11-444	*PKD1*	Yes	c.8041C>T	p.(Arg2681Cys)	Absent	0	Highly likely pathogenic	38	20
12-444	*PKD2*	–	c.1532_1533insAT	p.(Asp511fs)	Absent	0	Definitely pathogenic	156	70
	*PKD1*	–	c.10921C>T	p.(Arg3642Cys)	Absent	0	Highly likely pathogenic	40	52
12-505	*PKD1*	Yes	c.50174_5015delAG	p.(Arg1672fs)	Present	28	Definitely pathogenic	118	88
13-199	*PKD1*	Yes	c.7039delC	p.(Arg2347fs)	Absent	0	Definitely pathogenic	34	32
12-628	*PKD1*	Yes	c.2180T>C	p.(Leu727Pro)	Absent	0	Highly likely pathogenic	20	8
08-258	*PKD1*	Yes	c.7925C>T	p.(Arg2639*)	Present	5	Definitely pathogenic	28	14
10-484	*PKD1*	–	c.12010C>T	p.(Gln4004*)	Present	4	Definitely pathogenic	26	38
13-102	–	–	–	–	–	–	–	–	–
07-335	–	–	–	–	–	–	–	–	–

# patients in previous studies, NM_001009944.2 for *PKD1* and NM_000297.2 for *PKD2*.

## Discussion

It has been suggested that the impact that NGS technologies will have on clinical genetics during the upcoming years will be comparable to the introduction of X-rays to medicine many decades ago (Hennekam and Biesecker 2012). After the tremendous impact of NGS technologies in the discovery of disease-causing genes during the last 4 years, we are witnessing the introduction of these technologies for diagnostic applications, with the aim of rapidly revolutionize the field of genetic diagnostics, making it much more cost- and time-effective, advance accuracy, and point to unsuspected yet treatable conditions. The purpose of this study was to develop a cost-effective method for the molecular diagnostics of ADPKD applying targeted NGS. First, we validated the assay in a cohort of 36 previously characterized ADPKD patients in which we detected 35 out of 36 known mutations. Second, we analyzed a discovery cohort of uncharacterized ADPKD patients and we reached a diagnostic rate of 83% (10 out of 12 patients), allowing test reporting 5 days after receiving the DNA samples. Although the size of our cohort is modest, these results are very encouraging since these numbers represent a diagnostic rate comparable to data obtained by Sanger sequencing (Audrezet et al. 2012; Cornec-Le Gall et al. 2013) and NGS (Rossetti et al. 2012).

Recently, targeted sequencing by NGS has been used in the identification of mutations in ADPKD. Rossetti et al. (2012) did not apply a capture protocol for *PKD1* and *PKD2* enrichment since they speculated that the duplicated genomic regions of *PKD1* would lead to concurrent capture of the six *PKD1* pseudogenes making very difficult the identification of the ADPKD causal variants. Instead, these authors used a strategy of pooling equimolar LR-PCR amplicons and multiplexing barcoded libraries. Their approach showed a high sensitivity, specificity and accuracy, but it is a very laborious task more amenable to characterize large ADPKD populations than for routine genetic diagnosis. Moreover, their approach did not allow detecting large genomic rearrangements. Here, we do not only demonstrate that genome enrichment by in-solution hybridization using an elaborated probe design is an accurate strategy for mutation identification in the duplicated regions and the rest of *PKD1* and *PKD2*, but also that this strategy is ready to substitute LR-PCR-based methods in the routine genetic diagnostics of ADPKD to detect all sorts of sequence variants, including SVs.

When we conceived this study, we assumed that it would be extremely difficult to specifically capture the genuine *PKD1*, that is, there would always be residual enrichment of the six pseudogenes. Therefore, instead of excluding this region from our assay we decided to include unspecific probes to the duplicated region of *PKD1* in our capture library. In this regard, the mutation calls are on average lower than the reference calls, most likely due to the pseudogenes background (Tables 2, 3). From our point of view, the critical point of the assay was not the presence of sequencing reads coming from both the genuine *PKD1* and its pseudogenes. Instead, the major challenge was to map the reads coming from duplicated regions unambiguously to the genuine *PKD1* or to the six pseudogenes.

In order to minimize the impact of sequence reads coming from the pseudogenes we allowed mapping to the whole genome, instead of restricting the mapping to the targeted region. Moreover, the length of the millions of overlapping sequencing reads produced in this study (2 × 100 bp and 2 × 250 bp in the validation and discovery cohorts, respectively) in combination with the 300 bp insert sizes in the DNA libraries provided enough sequence specificity for accurate mapping and pseudogene discrimination, allowing us to unambiguously map a large proportion of the sequencing reads to *PKD1* (Table 1).

Furthermore, we also assume that the alignment algorithm is not 100% reliable and some reads coming from the pseudogenes could have been erroneously aligned to *PKD1*. In the worst scenario, the accumulation of these misaligned reads could lead to spurious variant calls but, as we have observed, none of these potential false positive variant calls passed the stringent filters of our variant prioritization pipeline in any of the patients of the validation and discovery cohorts neither in the five control samples.

The low sequencing coverage obtained for exons 1 and 42 of *PKD1* and exon 1 of *PKD2*, likely due to a high GC content, is the main limitation of this study as all variants that were missed were located in these poorly covered regions highlighting the importance of achieving sufficient depth of coverage for the optimal performance of the assay. However, we think that this can be fixed by rebalancing and adding new and replicate probes hybridizing with these poorly covered regions in the capture design. In the discovery cohort, the low average depth of coverage yielded by the MiSeq (Illumina, Inc.) in some samples was the cause of the lower mutation detection rate of the assay. However, we plan in the future to produce an optimized capture design including only *PKD1/PKD2* and a few cystic genes that would help in the differential diagnosis, such as *HNF1B* (17p12; OMIM ID: 189907) and *PKHD1* (6p12.3-p12.2; OMIM ID: 606702). This would significantly reduce the total captured DNA per sample, allowing multiplexing more samples per MiSeq (Illumina, Inc.) run, and to achieve higher depths of coverage (comparable to those obtained for the validation cohort) that will allow more confident variant calling.

We estimate that with our NGS-based assay a 60% of cost savings per sample could be achieved, and the whole diagnostics process could be a minimum of five times faster than with the conventional techniques currently used for the genetic diagnostics of ADPKD. In addition, our strategy offers a complete definition of the captured genes, without the need for stepwise testing anymore and having to choose which gene to sequence first, and is capable to detect large genomic rearrangements and deep intronic variants. In the discovery cohort, the complete process of library preparation, genomic enrichment, NGS using a MiSeq (Illumina, Inc.), and bioinformatics analysis was completed in 5 days after reception of the DNA samples.

In conclusion, we illustrate here the first successful study using in-solution hybridization enrichment coupled to NGS to detect ADPKD pathogenic mutations, both in the duplicated regions of *PKD1* and the rest of *PKD1* and *PKD2* genes. Our approach is cost- and time-effective, and meets the sensitivity and specificity criteria required for genetic diagnostics, providing NGS experimental and bioinformatics approaches ready to substitute classic molecular tools in routine genetic diagnostics of ADPKD.

## Supporting Information

**Table S1.** Target regions for capture of the *PKD1* and *PKD2* genes.

**Table S2.** Average coverage statistics of *PKD1* and *PKD2* exons in the validation cohort.

**Table S3.** By sample sequencing of quality control and coverage statistics in the validation cohort.

**Table S4.** By sample sequencing quality control and coverage statistics in the discovery cohort in sequencing of *PKD1* and *PKD2*.

**Table S5.** NGS of *PKD1* and *PKD2* versus genotyping calls in the five control samples.
